# Patient-specific modeling of hemodynamic characteristics associated with the formation of visceral artery aneurysms at uncommon locations

**DOI:** 10.3389/fcvm.2022.1008189

**Published:** 2022-09-29

**Authors:** Siting Li, Xiaoning Sun, Mengyin Chen, Tianxiang Ma, Xiao Liu, Yuehong Zheng

**Affiliations:** ^1^Department of Vascular Surgery, Peking Union Medical College Hospital, Chinese Academy of Medical Sciences and Peking Union Medical College, Beijing, China; ^2^Department of Vascular Surgery, State Key Laboratory of Complex Severe and Rare Diseases, Peking Union Medical College Hospital, Chinese Academy of Medical Science and Peking Union Medical College, Beijing, China; ^3^Key Laboratory for Biomechanics and Mechanobiology of Ministry of Education, School of Biological, Beijing Advanced Innovation Center for Biomedical Engineering, Science and Medical Engineering, Beihang University, Beijing, China

**Keywords:** visceral artery aneurysm, computational fluid dynamics, wall shear stress, mathematical modeling, computational tomography angiography

## Abstract

**Objective:**

Hemodynamic characteristics play critical roles in aneurysm initiation and growth. This study aims to explore the effect of common hemodynamic parameters on the formation of visceral artery aneurysms (VAAs), especially those from the pancreaticoduodenal arteries or other uncommon locations, using real patients’ models.

**Methods:**

Three-dimension vessel models of 14 VAAs from 13 patients were selected and constructed from computed tomography angiography (CTA) images. Aneurysms were manually removed to perform computational fluid dynamics (CFD) simulations of the models before aneurysm formation. Flow field characteristics were obtained and compared at the aneurysm forming and para-aneurysm areas. Aneurysm forming models were categorized into high-wall-shear stress (WSS) and low-WSS groups according to WSS value at aneurysm forming versus para-aneurysm areas.

**Results:**

Computational fluid dynamics analysis revealed that the high WSS group had significantly higher WSSmax (*P* = 0.038), higher time average WSS (TAWSS) (*P* = 0.011), higher WSS gradient (WSSG) (*p* = 0.036), as well as lower oscillatory shear index (OSI) (*P* = 0.022) compared to the low WSS group. Significant higher WSSmax (*P* = 0.003), TAWSS (*P* = 0.003), WSSG (*P* = 0.041) and lower OSI (*P* = 0.021) was observed at the aneurysm forming site compared to both upstream and downstream areas.

**Conclusion:**

Both local increase and decrease of WSS and WSS gradient were observed for the visceral artery aneurysm forming area. Computational fluid dynamics analysis could shed light on the pathogenesis of visceral artery aneurysms at uncommon vessel locations.

## Introduction

Visceral artery aneurysms (VAAs) are a set of vascular conditions affecting celiac artery (CA), superior and/or inferior mesenteric arteries (SMA and/or IMA) and their branches, the prevalence of which is estimated around 0.01-02% in the general population (1, 2). The most commonly involved visceral arteries are splenic arteries (60-80%), while the rest of visceral arteries (hepatic arteries (HA), SMA, CA, pancreaticoduodenal arteries (PDA), etc.) account for less than 40%. Although only comprising 5% of all abdominal aneurysms, VAAs are prone to underdiagnosis and have a mortality rate of 25% or more once ruptured (1). Furthermore, due to an often-asymptomatic nature, a quarter of VAAs reported are presented with rupture (3). Thus, the timely recognition and reliable monitoring of VAAs are crucial for any sequential interventions.

Considering etiology, true VAAs mainly arise from atherosclerotic nature, collagen vascular diseases, and other rare causes, while pseudoaneurysms are most commonly due to trauma or iatrogenic injury, local inflammatory, and infection (3). Besides clinical risk factors, changes in the regional hemodynamic states (i.e., portal hypertension) were found to be associated with the initiation of splenic artery aneurysms (4). Aneurysms of the rest of visceral arteries, however, were less studied because of their rarity. An interesting phenomenon has been observed on several occasions where stenosis of the celiac axis, mostly from median arcuate ligament syndrome, could result in an increased flow through the PDA and might be associated with the development of aneurysms (5, 6). However, the etiologies of VAA are complicated and still require investigation.

Image-based computational fluid dynamics (CFD) models of hemodynamics are powerful tools for studying a variety of cardiovascular diseases (7). Regarding aneurysms, a number of hemodynamic quantities have been proposed to be related to their growth or rupture in cerebral arteries or the aorta, such as wall shear stress (WSS), oscillatory shear index (OSI), gradient oscillatory number (GON), and aneurysm formation index (AFI) (8). Overall, CFD was less utilized in VAAs study, probably due to the rarity of these lesions. In this study, 14 PDA, SMA, or CA models deriving from patients’ CT images were applied to elucidate the role of hemodynamic parameters in VAAs initiation. Aneurysms were manually removed to mimic a hypothetically “initial” condition, where CFD analysis was performed and discussed.

## Materials and methods

### Patient and model acquisition

**FIGURE 1:**
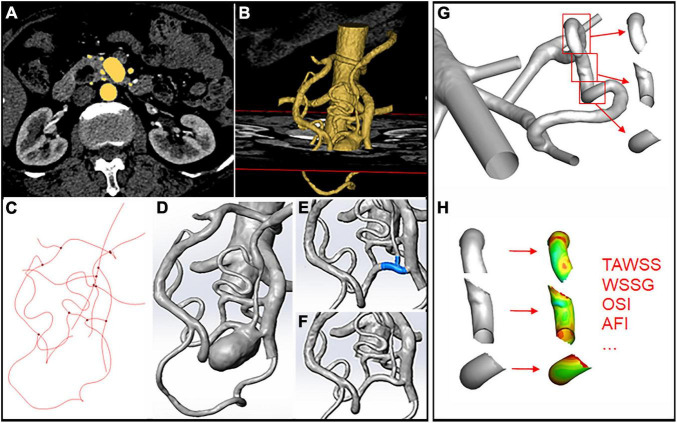
Schematic illustration of aneurysm model construction and hemodynamics data extraction. **(A,B)** 3D segmentation of the artery models through CTA images. **(C)** Centerline calculation in Mimics. **(D)** Artery model loaded into Solidworks after smoothing. **(E,F)** Removal of the aneurysm through centerline interpolation and connection of the remaining branches. **(G,H)** Segmentation of the upstream, downstream, and aneurysm areas using the “blanking” function and hemodynamic parameters.

We retrospectively examined patients with visceral aneurysms from Peking Union Medical College Hospital medical record database from 2013 to 2020. Patients who had splenic artery aneurysm or did not receive computed tomography angiography (CTA) examinations were excluded. Totally 13 patients with single or multiple aneurysms on the PDA, SMA, or CA were included in the study. As illustrated in Figure 1, CTA images were obtained and loaded into a commercial software Mimics (v21.0, Materialize) for 3D segmentation. Artery models of the aorta and main visceral branches (RA, CA, SMA, IMA, PDAA, and significant collateral branches) were extracted, with small branches manually excluded. Models were subsequently smoothed with Wrap (v2017, Geomagic) and loaded into Solidworks (v18, Solidworks Co.). Vessel outlets were trimmed and sufficiently extended to allow the fully developed flow pattern. Fourteen aneurysms were selected and removed from the models using a previously reported method (9). Briefly, the centerline of the model was first calculated, and the segment affected by the aneurysm was manually removed. The centerline was then interpolated and used to guide the interpolation of spheres between the remaining geometries, from which the parent artery surface was reconstructed.

### Computational fluid dynamics simulation

For each model, mesh convergence was investigated by comparing the WSSmax at peak systole with 6 meshes from coarse to fine. We calculated the percentage change in WSSmax, and a mesh was chosen when the percentage change was within 1%. Consequently, around 2 million (minimum mesh size of 1852566 and maximum mesh size of 2467559) finite volume tetrahedral elements were selected for each geometry using ANSYS ICEM (v19, ANSYS Inc.), and five layers of wall prism elements with the first layer of 0.05mm and a growth factor of 1.2 were added. Afterward, CFD simulations were performed with ANSYS Fluent (v19, ANSYS Inc.). Blood was modeled as an incompressible, Newtonian fluid with a density of 1,056 kg/m^3^ and viscosity of 0.0035 kg/ms. A rigid-wall no-slip boundary condition was implemented at the vessel walls. Inlet pressure and velocity profiles were derived from previous studies and adjusted with the cross-sectional area at each vessel outlet (10, 11). We performed a pulsatile flow simulation for 3 heart cycles to ensure convergence, and results from the last cycle were exported into Tecplot (Tecplot Inc.) for post-analysis.

### Hemodynamics analysis

The following hemodynamic variables were calculated and evaluated: wall shear stress (WSS), time average wall shear stress (TAWSS), wall shear stress gradient (WSSG), oscillatory shear index (OSI), and aneurysm formation index (AFI). Detailed definitions of these variables could be found in previous studies (8, 12, 13). Aneurysm forming area and para-aneurysm (upstream or downstream) areas were defined as vessel segments at and around the removed aneurysms. The accurate position of each area was identified and isolated using the “blanking” function in Tecplot (Figure 1C). Area average hemodynamic parameters were calculated for the aneurysm forming and para-aneurysm areas on the vessels. All the models were categorized into high-wall-shear stress (WSS) and low-WSS groups according to WSS value at aneurysm forming versus para-aneurysm areas.

### Statistical analysis

Two-tailed Student t-tests or Wilcoxon tests were used for cross or within-group comparison of hemodynamic parameters. The Chi-square test was used for categorical variables. Analyses were performed using IBM SPSS (v26, IBM Corp.), and *p* ≤ 0.05 was considered statistically significant.

## Results

### Patient and aneurysm characteristics

Totally 14 aneurysms from 13 patients were included in the study, and baseline characteristics were summarized in Table 1. Symptoms included abdominal pain or discomfort at disease onset. Hemodynamic parameters were calculated for each subject. The average WSS was 18.5 Pa for the high-WSS group and 3.5 Pa for the low-WSS group. No significant difference was observed between the two groups in baseline patient characteristics (age, sex, smoking habits, *etc.*) or aneurysm size. Regarding the location of the aneurysm, PDAA was associated with stenosis/occlusion of the SMA or CA (*p* = 0.031, Table 2).

**TABLE 1:** Baseline characteristics of patients and aneurysms.

Characteristics	High-WSS (*n* = 11)	Low-WSS (*n* = 3)	*P*
Age, years, mean (SD)	57.2 (15.5)	55.0 (4.4)	0.185
**Number of patients, N**			
Male sex (%)	6 (60)	2 (66.7)	1.0
Hypertension (%)	6 (60)	1 (33.3)	0.559
Smoking (%)	3 (30)	1 (33.3)	1.0
Symptom (%)	6 (60)	2 (66.7)	1.0
Aneurysm size, cm	5.6 (1.8)	5.5 (1.7)	0.693
**Aneurysm location, N**			
PDA	3	2	–
CA	1	1	–
SMA	6	0	–
**Hemodynamic parameters**			
WSS_*max*_, Pa	18.46 (13.71)	3.48 (0.81)	0.038*
TAWSS, Pa	1.31 (0.83)	0.26 (0.11)	0.011*
WSSG, N/m^3^	605.27 (524.93)	81.13 (37.52)	0.036*
OSI, *e^–7^	7.34 (6.43)	26.97 (11.23)	0.022*
AFI, m^2^/N	0.67 (0.29)	0.56 (0.23)	0.392

*P < 0.05.

**TABLE 2:** Association of aneurysm location with artery stenosis/occlusion.

		Stenosis/occlusion		Total	Fisher’s
Variable		0	1		*p*
Aneurysm Location	N-PDAA	6	3	9	0.031
	PDAA	0	5	5	
	Total	6	8	14	

### Hemodynamic difference between high-WSS and low-WSS groups

**FIGURE 2:**
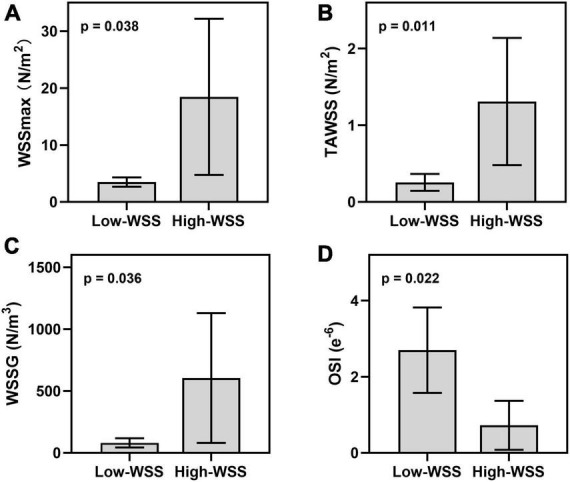
Hemodynamic difference between high-WSS and low-WSS groups in the aneurysms forming area. **(A)** Results of WSSmax. **(B)** Results of TAWSS. **(C)** Results of WSSG. **(D)** Results of OSI.

**FIGURE 3:**
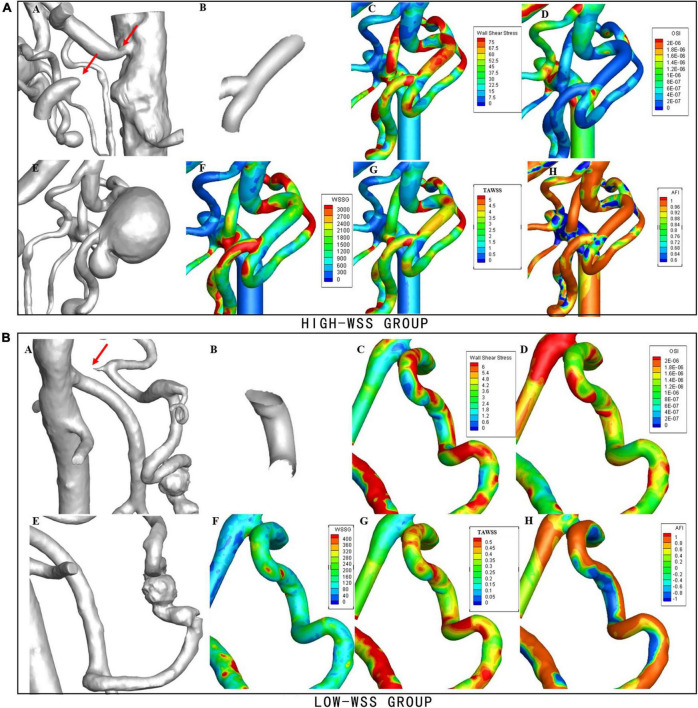
Examples of hemodynamic parameters in the high-WSS and low-WSS aneurysms. **(Upper panel)** AN example from the high-WSS group. **(A)** Concurrent stenosis of CA and occlusion of SMA. **(B)** Aneurysm forming area. **(C)** Results of WSS. **(D)** Results of OSI. **(E)** Aneurysm located at the bifurcation of PDA. **(F)** Results of WSSG. **(G)** Results of TAWSS. **(H)** Results of AFI. **(Lower panel)** An example from the low-WSS group. **(A)** Stenosis of CA. **(B)** Aneurysm forming area. **(C)** Results of WSS. **(D)** Results of OSI. **(E)** Aneurysm located on PDA. **(F)** Results of WSSG. **(G)** Results of TAWSS. **(H)** Results of AFI.

As illustrated in Figure 2, calculation of hemodynamic parameters indicated that the high-WSS group also had significantly higher WSSmax (*P* = 0.038), higher TAWSS (*P* = 0.011), higher WSSG (*p* = 0.036), as well as lower OSI (*P* = 0.022) than those of low-WSS group. Figure 3 gave an illustrative example of CFD results for each group.

### Hemodynamic characteristics in aneurysm forming and para-aneurysm area

Table 3 summarized the difference between aneurysm forming and para-aneurysm area in all aneurysms or each group. Wilcoxon tests were conducted to compare the results from the upstream aneurysm area, downstream aneurysm area, or their average with that in the aneurysm forming area, with *Z* and *P*-value shown. For all aneurysms in the high-WSS group, WSSmax and TAWSS were significantly higher at the aneurysm forming site compared to both upstream and downstream areas (*P* = 0.004 and 0.026). On average, a reduction of more than 30% percent WSS was present at the para-aneurysm area. Similar results were observed for the WSS-high group (*P* = 0.003). In addition, significantly higher WSSG and lower OSI were also observed in this group (*P* = 0.041 and 0.021). Little difference was present for the low-WSS aneurysms.

**TABLE 3:** Hemodynamic characteristics in aneurysm forming and para-aneurysm area among different groups.

		All (*n* = 14)			High WSS (*n* = 11)			Low WSS (*n* = 3)		
Variables		A vs Para	A vs Up	A vs Do	A vs Para	A vs Up	A vs Do	A vs Para	A vs Up	A vs Do
WSS_*max*_ (Pa)	Z	−2.919	−2.480	−2.856	−2.934	−2.934	−2.934	−0.535	−1.604	0.000
	*p*	0.004*	0.013*	0.004*	0.003*	0.003*	0.003*	0.593	0.109	1.000
TAWSS (Pa)	Z	−2.229	−1.350	−2.605	−2.934	−2.045	−2.756	−1.604	−1.604	0.000
	*p*	0.026*	0.177	0.009*	0.003*	0.041*	0.006*	0.109	0.109	1.000
WSSG (N/m^3^)	Z	−1.287	−0.659	−1.852	−2.045	−1.245	−1.956	−1.604	−1.069	−0.535
	*p*	0.198	0.510	0.064	0.041*	0.213	0.050*	0.109	0.285	0.593
OSI	Z	−0.973	−0.345	−1.852	−2.312	−1.600	−1.423	−1.604	−1.604	−1.069
	*p*	0.331	0.730	0.064	0.021*	0.110	0.155	0.109	0.109	0.285
AFI (m^2^/N)	Z	−1.412	−0.973	−0.659	−1.423	−0.889	−0.622	−0.535	0.000	0.000
	*p*	0.158	0.331	0.510	0.155	0.374	0.534	0.593	1.000	1.000

*P < 0.05.

## Discussion

VAA involving CA, SMA, and PDA is extremely rare yet highly susceptible to rupture (14). In our study, 9 of 13 (69.23%) patients included showed different degrees of aneurysm-related symptoms, probably because of admission bias. As for clinical factors, hypertension and smoking were prevalent in our patients, suggesting that atherosclerosis might influence these vessel models and favor aneurysm formation. Previous results from a large cohort indicated that most VAAs were asymptomatic, and more than half of them could remain stable under surveillance over time without size change or rupture (15). Thus, although the development of ultrasonography, CTA, MRI, and other diagnostic tools have increased the finding of asymptomatic VAAs, whether they should be referred to frequent surveillance or surgical intervention need finer assessment.

High WSS and low WSS are two key theories in aneurysm formation. Under normal conditions, endothelium cells (ECs) and smooth muscle cells (SMCs) on the arterial wall sense stress and shear stress through the cell membrane, primary cilia, integrin, and other functional components, triggering downstream signal transmission to maintain homeostasis. Supraphysiological high WSS could lead to increase MMP2 and MMP9 expression in ECs as well as loss of SMCs. Low WSS, on the other hand, may result in the increased expression of the pro-inflammatory factors and proliferation of inflammatory cells (16). OSI and AFI were parameters indicating fluctuation of WSS direction over the flow cycle, which could be sensitive for endothelial cells (12). Previous studies proposed that high WSS combined with a positive WSSG trigger a mural-cell-mediated destructive remodeling and growth of large aneurysm, while low WSS and a high oscillatory shear index can trigger inflammatory-cell-mediated destructive remodeling and formation of small bleb aneurysm (17).

In a meta-analysis, Can et al. concluded that an increase in WSS and GON may contribute to intracranial aneurysm formation, whereas low WSS was associated with its rupture (18). In another study, Huang et al. found a negative correlation between WSS and aneurysm for middle cerebral artery (MCA) bifurcation aneurysm (19). However, unlike intracranial aneurysms (20), VAA models are less common and most of the studies applying CFD analysis to VAAs are single or series case reports. These papers mainly focused on constructing ideal computational models, while true vessel models from VAA patients were less utilized. Mano et al. successfully used 4-D magnetic resonance imaging (MRI) to explore various hemodynamic parameters in PDAA pathogenesis (21).

Our study suggested that both high- and low- WSS mechanisms could play a role in the initiation of VAAs. For the high-WSS group, not only a higher WSS_*max*_ and TAWSS was observed at aneurysm forming area compared to para-aneurysm area, higher mean average level of WSS_*max*_ and its spatial gradient was also present than the low-WSS group. The result implied that for visceral artery at overall high WSS states (e.g., WSSmax > 18.46 Pa, TAWSS > 1.31 Pa, and WSSG > 605.27 N/m^3^, as calculated by the average value from this study), a local increase of WSS compared to the adjacent area indicated a high risk of aneurysm formation. For visceral artery at overall low states (WSSmax < 3.48 Pa, TAWSS < 0.26 Pa, and WSSG < 81.13 N/m^3^), local decrease of WSS were potential sites of aneurysm initiation. However, considering the heterogenous structures and the low number of models, the exact cut-off value should be explored with more patient-specific samples. More significant results were present between the aneurysm forming and the downstream area compared to the upstream area. This might be explained by an increase of blood velocity along the flow direction due to the decrease of vessel diameter. However, no significant difference was observed between the upstream and downstream aneurysm area (result not shown).

Notably, all PDAA included had various degrees of SMA or CA stenosis, which was consistent with previous findings (6). It had been reported that stenosis or occlusion of proximal intracranial arteries could lead to increase WSS at distal areas, which was associated with aneurysm formation (22, 23). Yuhn et al. investigated the hemodynamic characteristics of patients with CA stenosis and simulated arterial modeling of the PDA (24). Li et al. simulated the flow field WSS of a patient with PDAA accompanied by SMA occlusion to predict the outcome of different operation strategies (25). Additionally, Yonn et al. used an electric circuit model to explore the relationship between CA stenosis and PDA perfusion (26). Our study demonstrated that this condition could also be directly simulated with CFD analysis. On the other hand, significantly lower OSI was only observed for the high-WSS group. The role of OSI in aneurysm forming and rupture remains controversial (27).

Little association was found between the WSS states and the location or size of aneurysms, suggesting that the initiation and growth of aneurysms were not simply influenced by the primary blood flow rates or vessel diameters. Aneurysm progression could reversely influence local hemodynamics states, possibly ameliorate or accelerate its growth (26, 28). Nevertheless, since the time course of disease before diagnosis was neither available nor consistent for every patient, we could only remove all aneurysms and evaluate them at hypothetically “initial” conditions.

In summary, our series of case models pointed out some hemodynamic abnormities at VAA forming sites. In recent years, with the development of imaging technology, the application of CFD analysis to real 3D vessel models from patients is becoming a growing trend. Thus, it would be possible to have a preliminary evaluation of VAA forming potential for high-risk patients (for instance, those with collagen vascular diseases, identified aneurysms at other sites, or median arcuate ligament syndrome) using CTA results that were conveniently acquired in clinical scenarios. We were aware that the study had some limitations. For once, individual boundary flow conditions of vessels models were not available, which may influence the accuracy of calculation (20). PC-MRI or doppler ultrasound could be applied in the future to solve this problem. Another disadvantage was the limited number and heterogeneity of cases due to their low incidence. In the future, a similar study protocol could be applied to more homogeneous models or in a larger cohort to consolidate our findings, and patients’ flow waveforms should be collected beforehand. Furthermore, since the initiation of an aneurysm could be influenced by numerous factors, incorporating clinical risk factors could provide more predictive value in aneurysm diagnosis and assessment.

## Conclusion

In conclusion, both local increase or decrease of WSS and its special gradient were observed for the visceral artery aneurysm (VAA) forming area on the patients’ vessel models. Hemodynamic characteristics could shed light on the pathogenesis of rare VAAs.

## Data availability statement

The raw data supporting the conclusions of this article will be made available by the authors, without undue reservation.

## Ethics statement

The studies involving human participants were reviewed and approved by Ethics Committee of Peking Union Medical College Hospital. The patients/participants provided their written informed consent to participate in this study.

## Author contributions

SL, XS, XL, and YZ contributed to the design of the study. SL, XS, and MC contributed to data collection and performing simulation. SL contributed to statistical analysis. SL, XS, and TM contributed to the manuscript writing. All authors have read and approved the content and agreed to its submission.

## References

[1] van Rijn MJ Ten Raa S Hendriks JM Verhagen HJ. Visceral aneurysms: old paradigms, new insights? *Best Pract Res Clin Gastroenterol.* (2017) 31:97–104. 10.1016/j.bpg.2016.10.017 28395793

[2] Huang YK Hsieh HC Tsai FC Chang SH Lu MS Ko PJ. Visceral artery aneurysm: risk factor analysis and therapeutic opinion. *Eur J Vasc Endovasc Surg Off J Eur Soc Vasc Surg.* (2007) 33:293–301. 10.1016/j.ejvs.2006.09.016 17097898

[3] Chaer RA Abularrage CJ Coleman DM Eslami MH Kashyap VS Rockman C The Society for Vascular Surgery clinical practice guidelines on the management of visceral aneurysms. *J Vasc Surg.* (2020) 72(Suppl. 1):3s–39s. 10.1016/j.jvs.2020.01.039 32201007

[4] Ohta M Hashizume M Ueno K Tanoue K Sugimachi K Hasuo K. Hemodynamic study of splenic artery aneurysm in portal hypertension. *Hepatogastroenterology.* (1994) 41:181–4. 8056411

[5] de Perrot M Berney T Deléaval J Bühler L Mentha G Morel P. Management of true aneurysms of the pancreaticoduodenal arteries. *Ann Surg.* (1999) 229:416–20. 10.1097/00000658-199903000-00016 10077055 PMC1191708

[6] Antoniak R Grabowska-Derlatka L Nawrot I Cieszanowski A Rowiński O. Aneurysms of peripancreatic arterial arcades coexisting with celiac trunk stenosis or occlusion: single institution experience. *BioMed Res Int.* (2017) 2017:1645013. 10.1155/2017/1645013 28286755 PMC5327782

[7] Morris PD Narracott A von Tengg-Kobligk H Silva Soto DA Hsiao S Lungu A Computational fluid dynamics modelling in cardiovascular medicine. *Heart (British Cardiac Society).* (2016) 102:18–28. 10.1136/heartjnl-2015-308044 26512019 PMC4717410

[8] Shimogonya Y Ishikawa T Imai Y Matsuki N Yamaguchi T. Can temporal fluctuation in spatial wall shear stress gradient initiate a cerebral aneurysm? A proposed novel hemodynamic index, the gradient oscillatory number (GON). *J Biomech.* (2009) 42:550–4. 10.1016/j.jbiomech.2008.10.006 19195658

[9] Ford MD Hoi Y Piccinelli M Antiga L Steinman DA. An objective approach to digital removal of saccular aneurysms: technique and applications. *Br J Radiol.* (2009) 82(Spec No 1):S55–61. 10.1259/bjr/67593727 20348537

[10] Vignon-Clementel IE Alberto Figueroa C Jansen KE Taylor CA. Outflow boundary conditions for three-dimensional finite element modeling of blood flow and pressure in arteries. *Comput Methods Appl Mech Eng.* (2006) 195:3776–96. 10.1016/j.jvssci.2020.11.032 34258601 PMC8274562

[11] Lantz BM Foerster JM Link DP Holcroft JW. Regional distribution of cardiac output: normal values in man determined by video dilution technique. *AJR Am J Roentgenol.* (1981) 137:903–7. 10.2214/ajr.137.5.903 7027775

[12] Mantha A Karmonik C Benndorf G Strother C Metcalfe R. Hemodynamics in a cerebral artery before and after the formation of an aneurysm. *AJNR Am J Neuroradiol.* (2006) 27:1113–8. 16687554 PMC7975719

[13] Soulis J Fytanidis D Seralidou K Giannoglou G. Wall shear stress oscillation and its gradient in the normal left coronary artery tree bifurcations. *Hippokratia.* (2014) 18:12–6. 25125945 PMC4103034

[14] Pitton MB Dappa E Jungmann F Kloeckner R Schotten S Wirth GM Visceral artery aneurysms: incidence, management, and outcome analysis in a tertiary care center over one decade. *Eur Radiol.* (2015) 25:2004–14. 10.1007/s00330-015-3599-1 25693662 PMC4457909

[15] Corey MR Ergul EA Cambria RP English SJ Patel VI Lancaster RT The natural history of splanchnic artery aneurysms and outcomes after operative intervention. *J Vasc Surg.* (2016) 63:949–57. 10.1016/j.jvs.2015.10.066 26792545

[16] Diagbouga MR Morel S Bijlenga P Kwak BR. Role of hemodynamics in initiation/growth of intracranial aneurysms. *Eur J Clin Investigat.* (2018) 48:e12992. 10.1111/eci.12992 29962043

[17] Meng H Tutino VM Xiang J Siddiqui A. High WSS or low WSS? Complex interactions of hemodynamics with intracranial aneurysm initiation, growth, and rupture: toward a unifying hypothesis. *AJNR Am J Neuroradiol.* (2014) 35:1254–62. 23598838 10.3174/ajnr.A3558 PMC7966576

[18] Can A Du R. Association of hemodynamic factors with intracranial aneurysm formation and rupture: systematic review and meta-analysis. *Neurosurgery.* (2016) 78:510–20. 10.1227/NEU.0000000000001083 26516819

[19] Huang Z Zeng M Tao WG Zeng FY Chen CQ Zhang LB A hemodynamic mechanism correlating with the initiation of MCA bifurcation aneurysms. *AJNR Am J Neuroradiol.* (2020) 41:1217–24. 10.3174/ajnr.A6615 32554419 PMC7357637

[20] Saqr KM Rashad S Tupin S Niizuma K Hassan T Tominaga T What does computational fluid dynamics tell us about intracranial aneurysms? A meta-analysis and critical review. *J Cereb Blood Flow Metabol Off J Int Soc Cereb Blood Flow Metab.* (2020) 40:1021–39. 10.1177/0271678X19854640 31213162 PMC7181089

[21] Mano Y Takehara Y Sakaguchi T Alley MT Isoda H Shimizu T Hemodynamic assessment of celiaco-mesenteric anastomosis in patients with pancreaticoduodenal artery aneurysm concomitant with celiac artery occlusion using flow-sensitive four-dimensional magnetic resonance imaging. *Eur J Vasc Endovasc Surg Off J Eur Soc Vasc Surg.* (2013) 46:321–8. 10.1016/j.ejvs.2013.06.011 23880423

[22] Shakur SF Alaraj A Mendoza-Elias N Osama M Charbel FT. Hemodynamic characteristics associated with cerebral aneurysm formation in patients with carotid occlusion. *J Neurosurg.* (2018) 130:917–22. 10.3171/2017.11.JNS171794 29726778

[23] Kono K Fujimoto T Terada T. Proximal stenosis may induce initiation of cerebral aneurysms by increasing wall shear stress and wall shear stress gradient. *Int J Numerical Methods Biomed Eng.* (2014) 30:942–50. 10.1002/cnm.2637 24706583

[24] Yuhn C Hoshina K Miyahara K Oshima M. Computational simulation of flow-induced arterial remodeling of the pancreaticoduodenal arcade associated with celiac artery stenosis. *J Biomech.* (2019) 92:146–54. 10.1016/j.jbiomech.2019.05.043 31202524

[25] Li D Ma J Wei C Zhao J Yuan D Zheng T. Hemodynamic analysis to assist treatment strategies in complex visceral arterial pathologies: case reports and discussion from pancreaticoduodenal artery aneurysm with superior mesenteric artery occlusion. *Ann Vasc Surg.* (2019) 59:308.e1–8. 10.1016/j.avsg.2019.02.049 31075464

[26] Yoon HJ Choi JS Shin WY Lee KY Ahn SI. Causal relationship between celiac stenosis and pancreaticoduodenal artery aneurysm: interpretation by simulation using an electric circuit. *BioMed Res Int.* (2020) 2020:2738726. 10.1155/2020/2738726 32596287 PMC7298276

[27] Jirjees S Htun ZM Aldawudi I Katwal PC Khan S. Role of morphological and hemodynamic factors in predicting intracranial aneurysm rupture: a review. *Cureus.* (2020) 12:e9178. 10.7759/cureus.9178 32802613 PMC7425825

[28] Tanoue T Tateshima S Villablanca JP Viñuela F Tanishita K. Wall shear stress distribution inside growing cerebral aneurysm. *AJNR Am J Neuroradiol.* (2011) 32:1732–7. 10.3174/ajnr.A2607 21984256 PMC7965397

